# Sex differences in the use of mechanical ventilation in a neurointensive care population: a retrospective study

**DOI:** 10.1186/s12890-024-03094-7

**Published:** 2024-06-18

**Authors:** Federica Stretti, Didar Utebay, Stefan Yu Bögli, Giovanna Brandi

**Affiliations:** 1 Institute of Intensive Care Medicine, University Hospital Zurich, Rämistrasse 100, Zürich, 8091 Switzerland; 2 Department of Neurology, Clinical Neuroscience Center, University Hospital Zurich, Frauenklinikstrasse 26, Zürich, 8091 Switzerland; 3 Universitätsspital Zürich - Neurointensivstation, Rämistrasse 100, Zürich, 8091 Switzerland

**Keywords:** Sex, Gender medicine, Neurocritical care, Invasive mechanical ventilation

## Abstract

**Background:**

In the general intensive care unit (ICU) women receive invasive mechanical ventilation (IMV) less frequently than men. We investigated whether sex differences in the use of IMV also exist in the neurocritical care unit (NCCU), where patients are intubated not only due to respiratory failure but also due to neurological impairment.

**Methods:**

This retrospective single-centre study included adults admitted to the NCCU of the University Hospital Zurich between January 2018 and August 2021 with neurological or neurosurgical main diagnosis. We collected data on demographics, intubation, re-intubation, tracheotomy, and duration of IMV or other forms of respiratory support from the Swiss ICU registry or the medical records. A descriptive statistics was performed. Baseline and outcome characteristics were compared by sex in the whole population and in subgroup analysis.

**Results:**

Overall, 963 patients were included. No differences between sexes in the use and duration of IMV, frequency of emergency or planned intubations, tracheostomy were found. The duration of oxygen support was longer in women (men 2 [2, 4] vs. women 3 [1, 6] days, *p* = 0.018), who were more often admitted due to subarachnoid hemorrhage (SAH). No difference could be found after correction for age, diagnosis of admission and severity of disease.

**Conclusion:**

In this NCCU population and differently from the general ICU population, we found no difference by sex in the frequency and duration of IMV, intubation, reintubation, tracheotomy and non-invasive ventilation support. These results suggest that the differences in provision of care by sex reported in the general ICU population may be diagnosis-dependent. The difference in duration of oxygen supplementation observed in our population can be explained by the higher prevalence of SAH in women, where we aim for higher oxygenation targets due to the specific risk of vasospasm.

## Introduction

Sex differences in the provision of care in critically ill patients have been well documented in the recent literature and they could result in the observed differences in outcomes by sex [1–5]. Overall, women are less likely to be admitted to the intensive care unit (ICU) than men [1, 6–8] and their duration of stay in ICU is shorter [9]. Women, and above all young women, are less likely to receive ICU treatment regardless of disease severity [2]. In particular, women are less likely than men to receive invasive mechanical ventilation (IMV) [7, 9–11], and the ventilation received is less protective [12]. Current data are insufficient to clarify the extent to which such inequalities originate from sex differences in pathophysiology (e.g., presentation of symptoms), risk stratification (diagnostic bias), treatment preferences or in use of resources.

In the general-ICU population, these disparities in IMV between sexes have been well documented, while in neurocritical care these have been less investigated. In the general ICU, patients undergo IMV primarily due to acute cardio-pulmonary failure or acute on chronic cardio-pulmonary failure [13]. Conversely, in neurocritical care, IMV is initiated in most cases due to respiratory failure secondary to central neurological or neuromuscular diseases or airway protection [14]. A different provision of IMV by sex in this population has to be investigated, as it might contribute to the observed sex-related differences in mortality in neurocritical care [4].

Data on sex related differences in IMV in neurocritical are missing. In order to fill this gap in the knowledge, this retrospective single center study investigates whether sex-related differences in use of IMV exist in neurocritical care patients, similarly to in the general ICU population. The primary aim of the study was to compare the frequency of use of IMV and other form of respiratory support between sexes, in order to identify possibilities to improve and personalize the treatment of these patients.

## Methods

### Study population

This retrospective study included all consecutive patients admitted to the neurocritical care unit (NCCU) of the University Hospital Zurich between January 2018 and August 2021.

Patients included were (1) adults (≥ 18 years old), (2) with neurological or neurosurgical main diagnosis as reason for the Neuro Critical Care Unit (NCCU)-admission, (3) NCCU-length of stay (LOS) more than 24 h (in order to exclude moribund patients or patients who were at the ICU for a short monitoring time post-surgery). The only exclusion criterion was patients’ written or documented oral refusal to have their data analyzed for research projects. The local ethic committee (Kantonale Ethikkommission Zürich, KEK) approved the study (BASEC 2022 − 00270, 22.03.2022), which was performed in accordance with the ethical standards as laid down in the 2013 Declaration of Helsinki. This manuscript adheres to the applicable STROBE guidelines.

### Data collection

Data was retrospectively collected from the prospective Swiss-ICU registry (MDSi- Minimal Dataset for ICUs) and, when not available in the MDSi, from the medical records of the included patients. Details of the MDSi dataset have previously been reported [15] and it includes administrative data (age, sex, weight, height, admission and discharge data, limitation of care) As well as clinical data (clinical diagnosis, severity scores, intensity of treatment, including the NEMS score) [16]. Neurological or neurosurgical main diagnosis were grouped as hemorrhagic stroke (aneurysmal subarachnoid hemorrhage (SAH), arteriovenous malformation, and spontaneous intracranial hemorrhage), ischemic stroke, traumatic brain injury, and other diagnoses (including infections, status epilepticus, and tumors). Data on limitation of care are assessed at the admission in the ICU based on an advance care directive, if available, and constantly reviewed with the next of kin during the ICU stay according to the presumed patients’ will.

The electronic medical records of the patients were further reviewed to retrieve data about intubation (planned vs. emergency), reintubation, tracheotomy, duration of mechanical ventilation or other forms of respiratory support (non-invasive mechanical ventilation (NIV) or High Flow Oxygen Therapy (HFOT)), presence of respiratory comorbidities or complications (single or multiple) as COPD/Asthma, heart failure, pneumonia, ARDS, or others. The duration of respiratory support is extrapolated as shifts (every shifts equals 8 h) and then converted to days.

### Statistical analysis

Statistical analysis was performed using SPSS version 26. Data was always dichotomized by sex (male vs. female). In a second step subgroup analysis were performed, where patients were stratified by age of patients (over vs. under 65 years of age, the working age population), diagnosis, modality of intubation (emergency or planned) or predicted mortality (Simplified Acute Physiology Score – SAPS II < 41, previously used in the literature [17, 18] as well as the median value in our cohort).

Descriptive statistics are reported as counts/percentages, mean ± standard deviation, or as median including the interquartile range (IQR) as appropriate. All continuous data were tested for normality using Shapiro–Wilk’s test. Categorical variables were compared with Pearson’s χ2 or Fisher’s exact test, continuous/ordinal variables using Student’s t-tests or Mann–Whitney U tests for parametric and non-parametric data, respectively, where appropriate. A p-value < 0.05 was considered statistically significant.

## Results

### Baseline characteristics

**Fig. 1:**
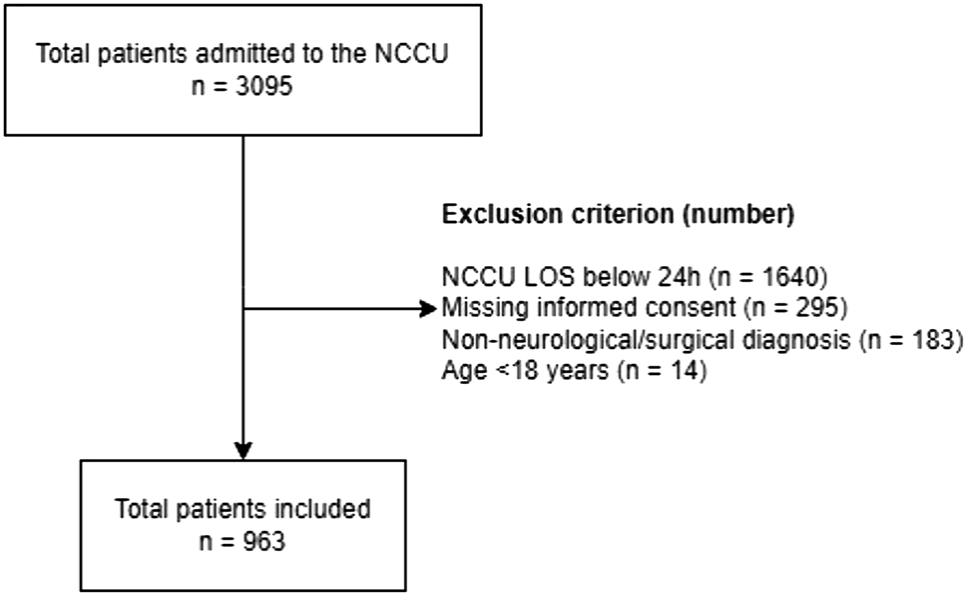
Flowchart on patients’ inclusion and exclusion

A total of 963 patients were included in the study, the majority of which were male (532 (55.2%) vs. 431 (44.8%), *p* = 0.001), as shown in Fig. 1. As outlined in Table 1, there was no difference in the baseline characteristics analyzed (BMI, age, SAPS, admission Sepsis-related Organ Failure Assessment – SOFA score, ward of origin of the patient), except for height and weight.

**Table 1:** shows the baseline characteristics of the 963 patients included in the study divided by sex. The statistically significant differences are highlighted

All Patients	Total	Male	Female	*p*-value
n (%)	963	532 (55.2)	431 (44.8)	**0.0013**
Age (years, mean ± SD)	59 ± 17	59 ± 17	60 ± 16	0.379
Weight (kg, mean ± SD)	75 ± 16	81 ± 17	69 ± 17	**< 0.001**
Height (cm, mean ± SD)	171 ± 10	175 ± 13	163 ± 10	**< 0.001**
BMI (kg/m^2^, mean ± SD)	26 ± 5	29 ± 31	28 ± 40	0.821
SOFA (median [IQR])	5 [3, 8]	5 [3, 9]	5 [3, 8]	0.129
SAPS (median [IQR])	32 [19, 48]	33 [19, 48]	32 [18, 48]	0.581
Origin of the patient				0.412
Operating room (n (%))	463 (48.1)	246 (46.2)	217 (50.3)	
Emergency department (n (%))	288 (29.9)	165 (31.0)	123 (28.5)	
Ward (n (%))	33 (3.4)	17 (3.2)	16 (3.7)	
ICU (n (%))	80 (8.3)	51 (9.6)	29 (6.7)	
Intermediate care unit (n (%))	99 (10.3)	53 (10.0)	46 (10.7)	
Emergency admission (n (%))	762 (79.1)	431 (81.0)	331 (76.8)	0.064
Diagnosis (n (%))				
Subarachnoid hemorrhage	280 (29.1)	116 (21.8)	164 (38.1)	**< 0.001**
Intracerebral hemorrhage	160 (16.6)	98 (18.4)	62 (14.4)	0.099
Subdural/epidural hemorrhage	17 (1.8)	12 (2.3)	5 (1.2)	0.227
Traumatic brain injury	23 (2.4)	19 (3.6)	4 (0.9)	**0.009**
Ischemic stroke	164 (17.0)	109 (20.5)	55 (12.8)	**0.002**
Cerebral tumor	126 (13.1)	59 (11.1)	67 (15.5)	**0.044**
Epilepsy	103 (10.7)	57 (10.7)	46 (10.7)	1
Neuromuscular disease	14 (1.5)	8 (1.5)	6 (1.4)	1
Infection	45 (4.7)	29 (5.5)	16 (3.7)	0.222
Other	64 (6.6)	37 (7.0)	27 (6.3)	0.698
Respiratory comorbidities (n (%))	387 (40.2)	225 (42.3)	162 (37.6)	0.078
Multiple respiratory comorbidities (n (%))	43 (4.5)	28 (5.3)	15 (3.5)	0.211
Pneumonia	342 (35.5)	194 (36.5)	148 (34.3)	0.499
COPD/Asthma	51 (5.3)	31 (5.8)	20 (4.6)	0.471
ARDS	5 (0.5)	3 (0.6)	2 (0.5)	1
Others (i.e. OSAS/ Pulmonary embolism)	1 (0.1)	6 (1.1)	0 (0.0)	**0.036**

SD – Standard Deviation, BMI – Body Mass Index, SOFA – Sequential Organ Failure Assessment, SAPS – Simplified Acute Physiology Score, ICU – Intensive Care Unit, COPD – Chronic Obstructive Pulmonary Disease, ARDS – Acute Respiratory Distress Syndrome, OSAS – Obstructive Sleep Apnea Syndrome

Women were more likely to be admitted to the NCCU for cerebral hemorrhage (men 245 (46.1%) vs. women 235 (54.5%), *p* = 0.010), in particularly SAH (men 116 (21.8%) vs. women 164 (38.1%), *p* < 0.001), and for tumors (59 (11.1%) vs. 67 (15.5%), *p* = 0.044). Men on the contrary were more likely to be admitted for ischemic stroke (109 (20.5%) vs. 55 (12.8%), *p* = 0.002) and traumatic brain injury (19 (3.6%) vs. 4 (0.9%), *p* = 0.009). There was no difference between sexes for other diagnosis at admission (subdural/epidural hematoma, epilepsy, neuromuscular disease, infection or other diagnosis).

### ICU management and treatment

**Fig. 2:**
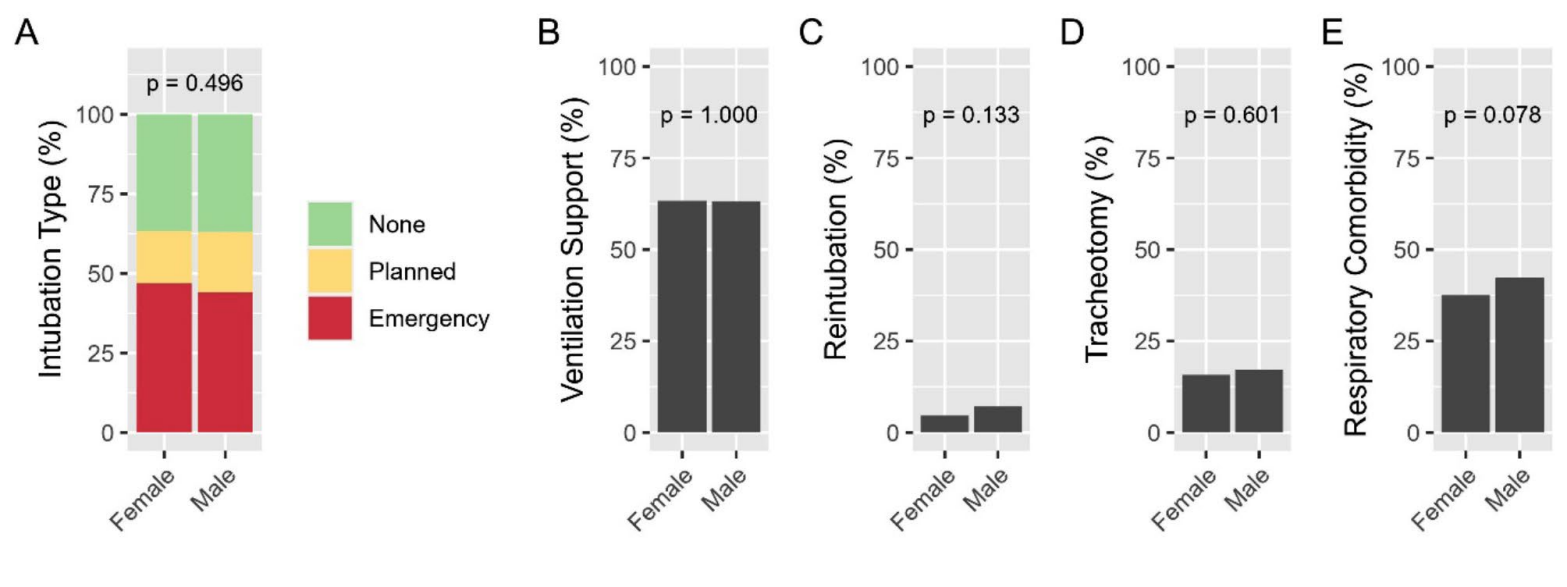
Comparisons between females and males on: **A** intubation type (none/ planned/ emergency); **B** frequency of ventilation support, **C** reintubation, **D** tracheotomy, and E frequency of respiratory comorbidities

**Fig. 3:**
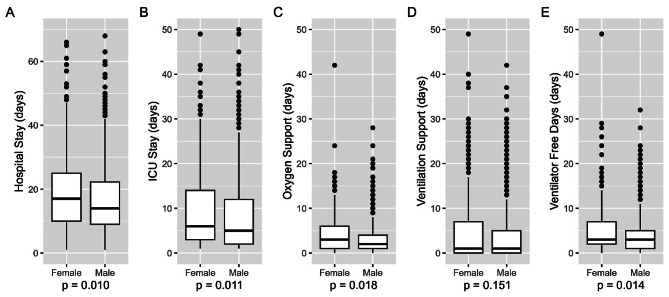
Comparisons between females and males on: **A** duration of hospital stay, **B** duration of neurocritical care unit stay, **C** duration of oxygen support, **D** duration of ventilation support, and E number of ventilator free days (all expressed in days)

Data are presented in Table 2; Figs. 2 and 3. There was no difference according to sex between emergency vs. non-emergency or no intubations (men 234 (44.0%) vs. women 202 (46.9%), *p* = 0.496, Fig. 2A). Overall, there were no difference between sexes regarding the number of invasively ventilated patients (men 336 (63.1%) vs. women 273 (63.3%), *p* = 1) (Fig. 2B) or subgroups concerning reintubation and tracheotomy (Fig. 2C and D). Men and women received invasive ventilation for the same number of days (men 1 [0, 5] vs. women 1 [0, 7] days, *p* = 0.151) (Table 2; Fig. 3D). On the contrary, there was a difference in the duration of oxygen support: women received oxygen for more days (men 2 [1, 4] vs. women 3 [1, 6], *p* = 0.018, Table 2; Fig. 3C). Women had a statistically significant longer ICU (10.0 ± 10 vs. 8.0 ± 9 days, *p* = 0.011) and hospital stay (20 ± 15 vs. 18 ± 13 days, *p* = 0.010, Fig. 3B) and more ventilator free days (Fig. 3E).

**Table 2:** ICU treatment and outcomes: ventilation support, length of stay and mortality shows the outcomes of the 963 patients included in the study divided by sex. The statistically significant differences are highlighted

	Male	Female	*p*-value
Intubation (n (%))	336 (63.1)	273 (63.3)	1
Intubation - emergency (n (%))	234 (44.0)	202 (46.9)	0.496
Reintubation (n (%))	38 (7.1)	20 (4.6)	0.133
Tracheotomy (n (%))	91 (17.1)	68 (15.8)	0.601
Ventilation support (days, median [IQR])	1 [0, 5]	1 [0, 7]	0.151
Oxygen support (days, median [IQR])	2 [1, 4]	3 [1, 6]	**0.018**
Ventilator free days (days, median [IQR])	2 [1, 5]	3 [1, 7]	**0.026**
LOS Hospital (days, mean ± SD)	18 ± 13	20 ± 15	**0.010**
LOS ICU (days, mean ± SD)	8 ± 9	10 ± 10	**0.011**
Limitation of care (n (%))	98 (18.4)	80 (18.6)	0.955
ICU mortality (n (%))	37 (7)	35 (8.1)	0.494

SD – Standard Deviation, LOS – Length of Stay, ICU – Intensive Care Unit

### Subgroup analysis

The subgroup analysis showed no difference and no trends in the outcomes according to severity (as expressed by SAPS), admission diagnosis, emergency vs. elective intubation, only invasively ventilated patients, age groups (Table 3). The difference in the duration of oxygen support was no more to be seen, especially in the subgroup of SAH patients, except in the young patients group, where the duration in oxygen support remained significantly longer for women.

**Table 3:** shows the subgroup analysis according to age, severity of disease and diagnosis at admission (only the results for subarachnoid hemorrhage are displayed here because it’s the most represented diagnosis in our cohort; all other analysis showed no significant differences)

Age	Male	Female	*p*-value
*Under 65 years old*	*n* = 321	*n* = 261	
Intubation (n (%))	195 (60.7)	153 (58.6)	0.611
Emergency intubation (n (%))	137 (42.7)	109 (41.8)	0.858
Reintubation (n (%))	20 (6.2)	14 (5.4)	0.724
Tracheostomy (n (%))	60 (18.7)	50 (19.2)	0.916
Non-invasive ventilation (n (%))	15 (4.7)	13 (5.0)	1
Oxygen support (days, median [IQR])	2 [1, 5]	3 [2, 6]	**0.026**
Ventilation support (days, median [IQR])	1 [0, 5]	1 [0, 7]	0.321
Ventilator free days (days, median [IQR])	3 [1, 6]	3 [2, 9]	**0.004**
*Over 65 years old*	*n* = 221	*n* = 170	
Intubation (n (%))	195 (60.7)	153 (58.6)	0.611
Emergency intubation (n (%))	137 (42.7)	109 (41.8)	0.858
Reintubation (n (%))	20 (6.2)	14 (5.4)	0.724
Tracheostomy (n (%))	60 (18.7)	50 (19.2)	0.916
Non-invasive ventilation (n (%))	15 (4.7)	13 (5.0)	1.000
Oxygen support (days, median [IQR])	2 [1, 4]	2 [1, 5]	0.343
Ventilation support (days, median [IQR])	2 [0, 5]	2 [0, 7]	0.277
Ventilator free days (days, median [IQR])	2 [1, 5]	2 [1, 5]	0.900
**Severity of disease**			
*SAPS ≥ 41*	*n* = 208	*n* = 162	
Intubation (n (%))	195 (93.8)	156 (96.3)	0.345
Emergency intubation (n (%))	146 (70.2)	124 (76.5)	0.330
Reintubation (n (%))	16 (7.7)	12 (7.4)	1.000
Tracheostomy (n (%))	56 (26.9)	44 (27.2)	1.000
Non-invasive ventilation (n (%))	18 (8.7)	12 (7.4)	0.705
Oxygen support (days, median [IQR])	2 [0, 4]	2 [0, 5]	0.158
Ventilation support (days, median [IQR])	3 [2, 10]	6 [2, 13]	0.113
Ventilator free days (days, median [IQR])	2 [0, 5]	2 [0, 6]	0.300
**Diagnosis at admission**			
*Subarachnoid hemorrhage*	*n* = 116	*n* = 164	
Intubation (n (%))	70 (60.3)	105 (64.0)	0.616
Emergency intubation (n (%))	50 (43.1)	86 (52.4)	0.222
Reintubation (n (%))	12 (10.3)	10 (6.1)	0.259
Tracheostomy (n (%))	24 (20.7)	29 (17.7)	0.539
Non-invasive ventilation (n (%))	11 (9.5)	7 (4.3)	0.089
Oxygen support (days, median [IQR])	6 [2, 10]	6 [2, 10]	0.916
Ventilation support (days, median [IQR])	1 [0, 6]	2 [0, 9]	0.428
Ventilator free days (days, median [IQR])	6 [1, 12]	6 [2, 12]	0.991

SD – Standard Deviation, SAPS - Simplified Acute Physiology Score

## Discussion

In our retrospective analysis of a NCCU population, differently from the general ICU population, we found no difference in the frequency and duration of invasive mechanical ventilation, intubation, reintubation, tracheotomy and non-invasive ventilation support between men and women. Women received longer duration of other forms of respiratory support and had a longer ICU and hospital stay, due to the higher prevalence of patients with SAH. These patients are monitored for a longer time and have higher oxygenation targets, due to the specific risk of vasospasm and delayed cerebral ischemia in the first two weeks after bleeding.

These findings suggest that ICU treatment differences by sex previously reported in the general ICU population could be at least partially explained by disease related pathophysiology and their specific management, as for example in SAH patients. Observed differences in the delivery of mechanical ventilation in ARDS and sepsis could be due to the wrong estimation of height and weight in women [11, 19].

### Ventilation

Differences in mechanical ventilation according to sex have been already reported and confirmed in a recent review and meta-analysis [3], also after adjustment for age and severity of illness. In the review, studies investigating individual diagnostic cohorts were excluded, leading to potential biases due to the missing differentiation of disease with specific characteristics and physiology. As an example, in the subgroup of sepsis patients, a recent meta-analysis found no difference in the delivery of ICU treatment [20]. Also in the group of burn patients, sex had no influence on ICU mortality [21]. On the contrary, there are more data regarding the reduced use of interventions and ICU resources for women with cardiovascular disease in comparison with men: women are less likely to receive a revascularization for cardiovascular disease also in Switzerland [2, 22]. It has also been described that women are less likely to receive a tracheostomy [23], but this was not the case in our case series.

We could demonstrate that in our cohort of patients women and men were ventilated with the same frequency, as concerns invasive and non-invasive methods of treatments. This inconsistency between our findings and the previous reports could reflect a complex interaction of physiological and sociocultural factors.

Firstly, to the best of our knowledge there are no studies specifically investigating the differences in mechanical ventilation based on sex in a neurosurgical ICU population. These patients have a peculiarity: they often require intubation because of neurological failure and not because of pulmonary failure. These findings suggest that at our NCCU the underlying neurological disease seems to have more relevance than the patient’s sex. Similarly, even if it was not the purpose of the article, Lioutas et al. [24] reported no sex difference in intubated vs. non intubated patients after intracranial hemorrhage.

The only difference we could find is in the duration of oxygen supplementation: overall more women have received oxygen supplementation in our NCCU patient population. At the same time, more women were admitted with a diagnosis of SAH. We postulate that this could be the reason for the longer supplementation of oxygen in women in general. In fact, in the subgroup analysis of patients with SAH, a difference is no more seen between sexes. There are well- known sex related differences in the epidemiology as well as treatment of SAH patients [25, 26]. Moreover, women have an higher risk of delayed cerebral ischemia in comparison to men [27], even though there is no difference in the development of an infarction or functional outcomes [28, 29]. Based on local protocols, higher partial pressure of oxygen (pO2) targets are aimed in patients with SAH to reduce the specific risk of delayed cerebral ischemia (DCI). Understanding the differences between sexes in the specific pathophysiology could, for example, lead to different pO2 targets: in women it could be necessary to aim for higher pO2 due to the specific higher risk of DCI.

Secondly, in this study we investigated patients admitted to a university hospital in a high-income country: in a situation where the resources are scarce, the sex-related biases could become more evident. Most data on sex differences in medicine come from higher income countries [3], where there could be now more awareness to possible sex-related biases. While our group reported difference regardless of severity score in the use of invasive disease specific treatments for intracranial bleeding (women received less insertions of external ventricular drainage despite same size of the ventricles and same neurology) [30], no differences where observed in our institution regarding treatment for intracranial tumors and SAH [31, 32]. The gender gap could have been narrowed thanks to guideline directed therapies [22].

### Length of stay in ICU

In our cohort, women had a significantly longer LOS in the ICU and in the hospital. Again, we can attribute the longer LOS in our ICU to the higher prevalence of women with a diagnosis of SAH: these patients are usually monitored for a longer time in our NCCU due to the risk of vasospasm in the first two weeks after presentation.

In contrast with previous works [33, 34], we did not find any difference in the prevalence of limitations of care [34]. We can then postulate that the absence of differences in the delivery of respiratory support between women and men in our cohort is not influenced by a different prevalence of limitation of care orders between man and women.

### Male predominance in the ICU

Consistent with findings from previous works [9, 35], there is a significant male predominance (532 (55.2%) vs 431 (44.8%), p = 0.001) also in our cohorts of patients in the NCCU. Differences in the triage process leading to ICU admission based on patients’ as well’ treating physicians’ sex have been described [36], leading to a disadvantage for women: men are more frequently admitted to the ICU in spite of the same severity of illness [6, 9, 37].

In our cohort of patients, we could not find any differences in the baseline characteristics. As already suggested previously [38], the male predominance in ICU patients could reflect the higher proportion of men with some neurological disease requiring ICU admission. In Switzerland, the incidence of stroke is in fact higher in men [39] and this could explain the higher proportion of men admitted to our ICU.

### Clinical implications

We assume that the inconsistency between our findings in a neurocritical care population and the previous reports referring to the general ICU population could reflect a complex interaction of physiological and sociocultural factors. Observed differences in the ICU treatment between sexes could be at least partially due to the disease specific pathophysiology and not to a discrimination, as previously postulated.

For the clinical practice and for future research, it is fundamental to understand first the role of sex in the disease epidemiology, presentation and pathophysiology. In a further step, it can be investigated the role that sex plays in the treatment choice. The disease itself, affecting differently the sexes, can lead to a different outcome and only conditionally lead to a different treatment.

### Limitations

This is a single center study in a high-income country, so these data and conclusion could not reflect that situation in other hospitals. Switzerland is a heterogeneous country with different nationalities and religions; our hospital is a referral center and our patients are representative of the Swiss population as far as religion is concerned (unpublished data, available upon request). We have no data on the respiratory status and vital parameters of patients at intubation and the reason for intubation (neurological failure vs. respiratory failure) is also unknown and this would be of interest for future research.

## Conclusions

In our cohort of patients admitted to the neurosurgical ICU, we could not find any difference in the provision of invasive respiratory support between sexes, differently from the general ICU population, where women are less often ventilated. In particular, we found no difference in the frequency and duration of IMV, intubation, reintubation, tracheotomy and non-invasive ventilation support between men and women. Women received longer duration of other forms of respiratory support, due to the higher prevalence of patients with SAH. Due to the specific risk of vasospasm in the first two weeks after bleeding, SAH patients are monitored for a longer time and have higher oxygenation targets.

## Data Availability

The datasets used and/or analysed during the current study are available from the corresponding author on reasonable request.

## Abbreviations

ICU: Intensive care unit
IMV: Invasive mechnical ventilation
NCCU: Neurocritical care unit
SAH: Subarachnoid hemorrhage
LOS: Length of stay
MDSi: Minimal dataset for ICUs
NIV: Non-invasive mechanical ventilation
HFOT: High flow oxygen therapy
SOFA: Sepsis-related organ failure assessment
